# The Effect of Physiotherapy on Dyspnea, Muscle Strength and Functional Status in Patients with Long COVID Syndrome

**DOI:** 10.3390/jpm14050515

**Published:** 2024-05-12

**Authors:** Michail Michalas, Stefanos Katsaras, Stavroula Spetsioti, Dimitrios Spaggoulakis, Archontoula Antonoglou, Andreas Asimakos, Paraskevi Katsaounou, Anna Christakou

**Affiliations:** 1 Department of Physiotherapy, School of Health Sciences, University of Peloponnese, 23100 Sparta, Greece; michailmichalas12@gmail.com (M.M.); stevekatsaras01@gmail.com (S.K.); 2 First Department of Critical Care Medicine, Evangelismos Hospital, Medical School, National & Kapodistrian University of Athens, 10676 Athens, Greece; roula_spe@hotmail.com (S.S.); dimitrisspglks@gmail.com (D.S.); arxoantonoglou@gmail.com (A.A.); silverakos@gmail.com (A.A.); paraskevikatsaounou@gmail.com (P.K.); 3 Department Physiotherapy, Lab Biomechanics, School of Health Sciences, University of Peloponnese, 23100 Sparta, Greece

**Keywords:** physiotherapy, muscle strength, functional status, dyspnea

## Abstract

Background: Patients who were infected with COVID-19 may experience Long COVID syndrome. We examined the effectiveness of physiotherapy on dyspnea, muscle strength, and functional status in Long COVID syndrome. Methods: The exercise group underwent an 8-week supervised physiotherapeutic program consisting of interval aerobic exercise and strengthening exercises, each lasting 30 min. The control group did not engage in any exercise. Dyspnea, muscle strength, and functional status were assessed at the beginning and end of the intervention in both groups. Results: No significant baseline differences were found between the two groups. The exercise group demonstrated improvements compared to baseline in dyspnea, quadriceps muscle strength, and functional status. Specifically, there was a significant increase of 3.7 lifts in the 60-s sit-to-stand test (*p* = 0.01), an increase of 5.86 kg in right quadriceps muscle strength (*p* = 0.03), an increase of 8.26 kg in left quadriceps muscle strength (*p* = 0.01), and a decrease in dyspnea score by 0.95 points (*p* = 0.02). Conclusions: Similar studies have reported improvements in dyspnea, muscle strength, and functional status in the exercise group. However, further research with larger sample sizes is needed to confirm these findings.

## 1. Introduction

The coronavirus disease 2019 (COVID-19) is known for causing respiratory illness, with patients exhibiting a wide range of symptoms during the acute stage of the disease, from mild to severe [1]. Following the initial acute infection, many patients experience a variety of long-lasting symptoms, even after testing negative for SARS-CoV-2 [2]. This condition, often referred to as “Long COVID Syndrome” is characterized by persistent or fluctuating symptoms lasting for weeks or months after the initial infection. These symptoms can include both physical and neuropsychiatric manifestations, for which no alternative diagnosis is found [3,4].

Patients with Long COVID syndrome present with a heterogeneous spectrum of systemic, cardiovascular, respiratory, gastrointestinal, musculoskeletal, neurological, neuromuscular, psychological, and other symptoms. Among these, the most frequently reported symptoms by patients include muscle weakness, fatigue, and shortness of breath [5]. Extensive research has demonstrated the negative impact of the syndrome on muscle strength [6,7,8], functional status [9,10,11,12,13,14] and dyspnea [15,16,17]. Predictors of Long COVID included Western ethnicity, BMI, chronic disease, COVID-19 reinfections, severity, symptoms, lower self-esteem, higher positive affect [18], age, and chest computed tomography severity score [19].

Treatment of patients with Long COVID syndrome involves a holistic approach, encompassing patient assessment, symptomatic treatment, management of comorbidities exacerbating symptoms, physiotherapy, and psychological support [20]. Physiotherapy has shown promise in improving muscle strength [21], functional ability, and dyspnea [22]. For instance, a study [22] reported significant enhancements in exercise capacity, functional status, dyspnea, fatigue, and quality of life among patients undergoing rehabilitation programs. Similarly, Araújo et al. [23] observed improvements in pulmonary ventilation, exercise capacity, fatigue, and quality of life with a combination of aerobic and resistance exercises. However, it’s noteworthy that some studies have reported exacerbation of symptoms due to exercise [24,25].

The aforementioned findings suggest that physiotherapy may be a promising method for treating the symptoms of Long COVID syndrome. However, there is limited research demonstrating the effectiveness of a physiotherapeutic exercise program incorporating escalating intensity and muscle load, which combines interval aerobic and resistance exercises, on functional status, dyspnea, and muscle strength in patients with Long COVID syndrome. Indeed, the existing literature on exercise prescription for Long COVID patients is relatively sparse [26]. More research is needed to provide a detailed description of physiotherapy programs tailored to this clinical population [27].

Therefore, the present study, which is the second part of our previous research project [28], aimed to examine the effect of a physiotherapy program that combines interval aerobic exercise on muscle strength, functional status, and dyspnea in patients with Long COVID syndrome. The research hypothesis was that physiotherapy improves the muscle strength, functional status, and dyspnea of patients with Long COVID syndrome.

## 2. Materials and Methods

### 2.1. Study Design

This was a prospective, interventional, non-randomized parallel assignment two-arm study. This study has been registered and approved by the Ethics Committee of the School of Health Studies of the University of Peloponnese (202/19-4-2023) and the Scientific Council of the General Hospital of Evagelismos (171/10-5-2023). The study was in agreement with the declaration of Helsinki Ethics principles. The detailed protocol of the present study has been published [28]. The participants received an allocation 1:1 to either a supervised 8-week exercise program (Exercise Group)—after a referral from the physician- or not (Control Group). Patients who could participate in the supervised rehabilitation program comprised the exercise group (*n* = 10), whereas patients who were unable to participate (due to logistical/transport issues) comprised the control group (*n* = 10). A first baseline assessment prior to the intervention period (pre) and at the final of the post-assessment (post) was conducted by the end of the intervention period [28].

### 2.2. Participants

This study consisted of 20 patients diagnosed with Long COVID syndrome, aged from 21 to 79 years old (M = 52.8 years, SD = 4.5 years), with 50% (*n* = 10) being females. This sample is entirely distinct from the participants in the first part of our previous research project [28]. Participants were divided into two groups: an exercise group and a control group. The exercise group comprised ten individuals (four men and six women), aged from 21 to 67 years old (M = 50.90 years, SD = 14.42 years), who underwent the same intervention physiotherapy protocol. The control group consisted of the remaining ten patients (six men and four women), aged from 28 to 79 years old (M = 54.70 years, SD = 4.76 years). Demographic data were collected during the initial appointment, and all participants were informed about the study procedures and signed a written informed consent form. Participants had the right to withdraw from the study at any time, and the publication of results was ensured to be anonymous. The detailed protocol of the present study has been published [28].

In order for the participants to be included in the study, they had to meet the following criteria: (a) Long COVID diagnosis. All patients had positive molecular testing for COVID-19 infection, a CAT score ≥ 10 [29] and/or a PCFS score ≥ 2 [30], using WHO score [31], (b) age from 21 years to 80 years, (c) able to understand written and spoken language and perform orders, (d) be ambulatory, (e) have not previously participated in an organized supervised exercise program, and (f) not require oxygen administration during exercise [28].

Exclusion criteria for the sample were: (a) the presence of multiple comorbidities that would prevent their participation in an exercise program, (b) those with acute or unstable chronic conditions, such as unstable cardiomyopathy, ischemic heart disease, uncontrolled hypertension, and uncontrolled chronic obstructive pulmonary disease, and (c) reinfection with COVID-19 midway via the intervention [28].

### 2.3. Instruments

The following instruments were used:

(a) Handheld Dynamometer.

The muscle strength of the quadriceps muscles was assessed with a K-Force Muscle Controller (Kinvent Company, Paris, France). The test was performed three times bilaterally, and the mean score of the three replicates was calculated to minimize the measurement errors [32,33]. These measurements are expressed in kilograms (Kg).

(b) 60-s Sit to Stand test (60 s-SST).

The 60-s Sit-to-Stand (60 s-STS) test was utilized to assess functional status and fall risk in the participants. During the test, participants were instructed to sit on a chair without using hand supports, keep their legs parallel (feet not touching the chair), and hang their arms loosely or place them on their hips [34]. The use of the upper limbs for assistance is not permitted during the test. Participants were then instructed to complete as many cycles from sitting to standing as possible within 60 s at a self-paced speed. The 60 s-STS test is commonly employed for assessing the functionality and lower limb ability in post-COVID-19 patients [35]. This test has demonstrated good reliability, with intra-class correlation coefficients (ICCs) ranging from 0.80 to 0.95 [36].

(c) Borg Dyspnea Scale.

The Borg Dyspnea Scale is a reliable scale that was used to evaluate the perceived sense of dyspnea [37,38]. It consists of a vertical scale marked 0–10. It is commonly used for post-COVID-19 patients [20]. It was used to assess the perceived dyspnea of participants at the end of 60 s-STS test.

### 2.4. Procedure

All participants in the exercise group adhered to a standardized program lasting 8 weeks, with a frequency of 2 sessions per week. This program comprised aerobic exercise on a cycle ergometer and strengthening exercises with resistance [28]. The aerobic exercise sessions lasted for 30 min and consisted of interval training, with participants alternating between 30 s of exercise and 30 s of rest. Throughout the aerobic exercise, the second author provided encouragement to maintain an upright posture and practice diaphragmatic breathing while maintaining a constant pedaling rate (rpm) on the cycle ergometer. Additionally, all participants in the exercise group used a pulse oximeter to monitor heart rate and oxygen saturation during aerobic exercise [28]. As perceived dyspnea, measured using the Borg scale, decreased to a value of 1–2 at the conclusion of the aerobic exercise, the intensity on the cycle ergometer was incremented by one level [28].

The resistance exercises in the program included both upper extremity strengthening exercises, such as chest presses, pull-ups, and chest fly, as well as lower extremity exercises, including knee extensions. Each strength training session consisted of 3 sets of 10 repetitions for each exercise. As participants demonstrated proficiency in performing the exercises correctly and completed three sets of ten repetitions, the resistance level was increased [28]. During the strengthening exercises, the participants practiced diaphragmatic breathing by coordinating their breathing with the movements: inhaling during the pull phase of the exercise and exhaling during the descent phase. After completing each set, participants rested for a few seconds before commencing the next set. The control group did not engage in any exercise [28]. Muscle strength, functional status, and dyspnea were evaluated for both groups before and after the physiotherapy program by the first author, while the second author supervised the participants during the exercise physiotherapy program [28].

### 2.5. Statistical Analysis

A homogeneity test, using the independent samples *t*-test analysis, was performed on all variables of the study and the demographic characteristics of the two groups. A normality test of all variables was performed using the “Shapiro–Wilk test”. Descriptive statistics tests were performed using the means and standard deviations of the sample. One-way ANOVA analysis of variance and Mann–Whitney U test were performed to compare the two groups. Paired samples *t*-tests and Wilcoxon Signed Ranks Test were performed to examine any differences between the two measurements at the beginning and at the end in each group. Data analysis was performed using the Statistical Package for the Social Sciences (SPSS 29.00) with a statistical significance level of α = 0.05.

## 3. Results

Table 1 shows the demographic and clinical characteristics of the participants. No significant differences were found between the two groups (a) at baseline in any demographical and clinical characteristics and (b) on the time from acute infection to the inclusion of the study (control’s group inclusion time M (SD) = 101.80 (5.67) days, exercise’s group inclusion time M (SD) = 100.60 (5.33), t = 0.48, *p* = 0.63). There were no adverse events during the training sessions. Among participants in the exercise group, none left the training program.

The measurements of the (a) muscle strength of the right and left quadriceps and the (b) number of rises of the 60 s sit-to-stand test were normally distributed. Therefore, One-Way ANOVA analysis and paired samples *t*-test were performed to examine the differences between the two groups and between the two measurements of each group. The measurements of the dyspnea at the end of 60 s-SST were not normally distributed. Therefore, the Man-Whitney U and Wilcoxon signed-rank tests were performed to investigate the differences between the two groups and between the two measurements of each group.

One-way ANOVA analysis and Mann–Whitney U test showed no statistically significant differences for the variables of muscle strength, functional status, and dyspnea between the two groups at baseline and final measurements.

The results of paired *t*-tests showed significant improvements in muscle strength and in the number of rises during the 60 s-SST for the exercise group. Particularly, there was an improvement in muscle strength by an increase of 5.86 kg for the right quadricep muscle (28.06 ± 5.91 kg vs. 33.92 ± 6.49 kg, t = −2.17, *p* = 0.03) and 8.26 kg for the left quadricep muscle (26.06 ± 6.79 kg vs. 34.32 ± 7.58 kg, t = −2.83, *p* = 0.01) while for the functional status there was by an increase of 3.7 rises for the number of rises during the 60 s-SST (24.10 ± 7.18 rises vs. 27.80 ± 4.13 rises, t = −2.75, *p* = 0.01). The results of the Wilcoxon signed ranks test showed improvements for the exercise group by a reduction of 0.95 units for the perceived dyspnea at the end of 60 s-SST (2.50 ± 1.65 vs. 1.55 ± 0.89, z = −2.26, *p* = 0.02). The results of pre- and post-test statistical analyses for the exercise group are shown in Table 2.

Paired *t*-test and Wilcoxon signed ranks test analyses showed no statistically significant differences between pre- and post- for the control group in the muscle strength of the right or left quadriceps muscles, neither in the perceived feeling of dyspnea at the end of 60 s-SST. However, paired *t*-test analyses showed a significant improvement only in the number of rises at the end of the 60 s-SST by an increase of 3.7 rises (22.40 ± 3.37 vs. 26.10 ± 5.95, t = −2.79, *p* = 0.01). The results of pre- and post-test statistical analyses for the control group are shown in Table 3.

## 4. Discussion

The present study was designed to evaluate the effectiveness of a physiotherapy program that combines aerobic and resistance exercise, with a progressive increase in intensity and muscle load, on muscle strength, functional recovery, and dyspnea in patients with Long COVID syndrome. The results showed no significant differences between the exercise and control group in quadriceps muscle strength in both lower limbs. This may be due to the limited sample size. Additionally, the elderly are at increased risk of developing musculoskeletal symptoms during Long COVID, possibly due to the combined effect of the viral infection, the pre-existing decline in muscle mass, and the age-related function [39,40]. The observation that the majority of participants in the present study were elderly individuals is an important consideration when interpreting the results, as age-related factors may have influenced the outcomes. Elderly individuals often experience declines in muscle mass and strength, which can affect their response to rehabilitation interventions. Additionally, the pathophysiology of Long COVID syndrome may interact differently with age-related changes, potentially impacting the effectiveness of treatment approaches. The findings mentioned from a previous study [8] reporting ongoing muscle weakness in some Long COVID patients at 6 months post-infection further highlight the complex nature of the condition and the challenges in addressing muscular strength deficits. These findings underscore the importance of continued research efforts to better understand the long-term effects of COVID-19 on muscle health and to develop targeted rehabilitation strategies. On the contrary, Jimeno-Almazán et al. [41] showed improvements in lower body muscle strength with a larger sample size; suggesting that there may be variability in treatment responses among individuals with Long COVID syndrome. This underscores the need for further investigation to identify factors that contribute to treatment outcomes and to refine rehabilitation protocols accordingly.

The significant increase in left and right quadriceps strength observed in the exercise group following the intervention is an encouraging finding and suggests that the physiotherapy program had a positive impact on muscle strength in patients with Long COVID syndrome. This result is consistent with previous case studies by Mayer et al. [42] and Santos and Flores [43], which reported improvements in muscle strength following similar exercise interventions. The agreement between the findings of the present study and the case studies by Mayer et al. and Santos and Flores provides further support for the effectiveness of physiotherapy programs in improving muscle strength in individuals with Long COVID syndrome. These results highlight the potential benefits of incorporating both aerobic and anaerobic exercise components, as well as emphasizing the importance of tailored rehabilitation programs to address the specific needs of Long COVID patients. While the exercise group demonstrated significant improvements in muscle strength, the control group did not show similar gains. This further underscores the importance of structured exercise interventions in promoting recovery and addressing the persistent symptoms associated with Long COVID syndrome.

The lack of significant differences between the exercise and control groups in the 60 s-SST functional test contrasts with findings from the study by Jimeno-Almazán [44]. This disparity in results may be attributed to several factors, including differences in the frequency of exercise sessions and the inclusion of specific components in the rehabilitation program. The lower frequency of exercise sessions in the present study, with sessions occurring twice per week, may have contributed to the lack of significant improvements in functional status compared to studies with higher exercise frequencies. Additionally, the absence of balance exercises and verbal feedback in the rehabilitation program could have negatively impacted the outcomes, as these components are known to play crucial roles in enhancing functional abilities and motor skill acquisition. Despite the lack of significant group differences, the exercise group in the present study demonstrated a significant improvement in the number of rises in the 60 s-SST following the physiotherapy program. This finding suggests that the intervention may have had a positive effect on specific aspects of functional status, particularly lower limb strength and endurance. Consistent with these results, previous studies by Nopp et al. [22] and Mayer et al. [42] reported increases in the number of repetitions performed in similar functional tests following rehabilitation interventions. The control group in the present study did not show similar improvements in the 60 s-SST, highlighting the potential benefits of structured exercise interventions for enhancing functional status in individuals with Long COVID syndrome. However, further research is needed to explore the specific components of rehabilitation programs that contribute to improvements in functional status and to optimize treatment protocols for this patient population.

The absence of statistically significant differences in subjective dyspnea between the exercise and control groups in the present study contrasts with the findings of Jimeno-Almazán et al. [41]. Jimeno-Almazán et al. employed a larger sample size, randomized allocation, and a higher frequency of intervention sessions (3 sessions/week) compared to the present study. Additionally, their intervention included concurrent training with or without inspiratory muscle training, whereas the present study focused on a combination of aerobic and resistance exercises. These differences in experimental design and interventions could have influenced the outcomes related to dyspnea. Despite the lack of significant differences between groups, the exercise group in the present study demonstrated a significant reduction in dyspnea following the physiotherapy program, as indicated by changes in Borg Scale ratings. This finding aligns with results reported by Nopp et al. [22], who also observed a decrease in dyspnea following a rehabilitation program for individuals with Long COVID syndrome. These results suggest that structured physiotherapy programs may contribute to improvements in dyspnea among individuals with Long COVID syndrome, highlighting the potential benefits of such interventions for respiratory function and symptom management. Overall, while the present study did not find significant differences in dyspnea between groups, the observed reduction in dyspnea within the exercise group underscores the importance of exercise-based interventions in the management of respiratory symptoms in individuals with Long COVID syndrome.

The role of physical exercise in managing and rehabilitating Long COVID syndrome is still being elucidated [43], but evidence suggests its potential benefits in preserving functionality and reducing disability [44]. Exercise has demonstrated positive effects in alleviating severe COVID-19 symptoms, indicating potential benefits for individuals with Long COVID syndrome. Cardiopulmonary rehabilitation, including moderate continuous aerobic training and resistance training, has been shown to improve exercise tolerance, reduce fatigue, and positively impact psychological factors such as perceived quality of life [23]. As such, appropriate and personalized physiotherapy is emerging as a complementary treatment to alleviate persistent COVID-19 symptoms and expedite recovery [45].

Exercise training has shown potential in improving various objective measurements of Long COVID disease severity, such as V˙O2peak, V˙E/VCO2, fatigue, and 6 min walk distance, as well as subjective metrics like dyspnea and fatigue [22,23,41,44]. When designing exercise interventions for patients with Long COVID syndrome, it is crucial to consider the specific type of exercises to be administered, as well as tailoring criteria such as training loads, frequencies, intensities, and durations. It’s important to note that COVID-19 can lead to frequent fatigue and neurocognitive symptoms, which significantly impact the quality of life for individuals with Long COVID syndrome [46]. Physical exercise, while recommended for rehabilitation, can sometimes exacerbate symptoms such as post-exertion malaise [46] or have varying effects that can either improve or worsen symptoms [24].

The exercise programs that combine aerobic and anaerobic exercise [41,44], inspiratory muscle training [22,41], and diaphragmatic breathing [42] contributed to significant improvements in the exercise group after inspiratory muscle training programs [47]. On the contrary, when inspiratory muscle training was only applied, no significant improvement was reported for the intervention group compared to the control group [41,48]. Respiratory physiotherapy, as well as aerobic and resistance training, have shown promise in reducing dyspnea, fatigue, and sarcopenia, which are common symptoms of Long COVID-19 syndrome [27]. Cattadori et al. [26] proposed a multi-component exercise protocol that incorporates various exercises, including aerobic continuous and interval training (such as walking and cycling), resistance/strength training for both upper and lower body, inspiratory muscle training using handheld resistance tools, cough exercises, diaphragmatic muscle training in the supine position, stretching and mobilization, balance and flexibility exercises (both static and dynamic), and slow breathing exercises [26,49,50,51].

Following COVID-19, official guidelines for exercise training reported the value of beginning with low-intensity activities and progressively raising the intensity [48,52]. Taking into account the possible risks associated with intense exercise for individuals suffering from post-viral chronic fatigue syndrome, we employed intermittent exercise training on a cycle ergometer. After being released from the hospital, intermittent exercise has been demonstrated to be safe and well-tolerated by COVID-19 survivors [53,54]. For patients with respiratory disorders characterized by exertional dyspnea, intermittent exercise—which alternates brief intervals of exercise with periods of rest—has been shown to be beneficial [55]. Due to its association with mild exertional symptoms, intermittent exercise may, therefore, be more appropriate for COVID-19 survivors than continuous exercise [56]. Furthermore, during the exercise training program, no negative outcomes were reported, highlighting the safety of progressively increasing the intensity of intermittent exercise for this specific population during the rehabilitation process.

The study’s acknowledgment of its limitations is crucial for interpreting its findings and informing future research directions. The highlighted limitations provide valuable insights into potential factors that may have influenced the study’s outcomes and suggest avenues for improvement in future investigations. The main limitation is the small sample size. Beyond the participants’ willingness, difficult transportation to the hospital and a pessimistic view of the benefits of rehabilitation played a significant role in participants’ decision to take part or not. It has long been acknowledged that these variables play a major role in participants’ refusal of pulmonary rehabilitation [57]. Another limitation is that no longer-term follow-up was done, which would have yielded more data regarding the possible long-term impacts of physiotherapy rehabilitation. Additionally, we used pulse oximetry to measure HR; thus, future studies should utilize more valid measures to examine the HR of the sample. Conducting further research with controlled studies and larger sample sizes would greatly enhance our understanding of the effectiveness of physiotherapy interventions for managing Long COVID syndrome. Long-term intervention programs and follow-up assessments are crucial for evaluating the sustained benefits of exercise programs on muscle strength, functional status, and dyspnea over time. By addressing these aspects, future studies can provide valuable insights into the optimal rehabilitation approaches for mitigating the negative functional outcomes associated with Long COVID syndrome.

## 5. Conclusions

The findings suggest that a physiotherapy program can lead to improvements in muscle strength, dyspnea, and functional status in patients with post-COVID-19 syndrome compared to those who did not undergo the program. Furthermore, intermittent exercise training has been shown to be safe and effective in this population. These results align with similar studies that have reported improvements in patients who participated in exercise programs following COVID-19. However, it’s important to acknowledge the limitations of the present study, particularly the small sample size. Future research with larger sample sizes should be conducted to validate and further explore the findings of the present study. Ongoing research should continue to investigate the effectiveness of physiotherapy interventions in improving outcomes for patients with post-COVID-19 syndrome.

## Figures and Tables

**Table 1:** Characteristics of the participants.

Variables	Control Group (*n* = 10)	Exercise Group (*n* = 10)
Age, years M (SD)	54.70 (15.04)	50.90 (14.42)
Women, *n* (%)	4 (40)	6 (60)
BMI, kg/m^2^ M (SD)	27.46 (5.74)	26.58 (6.66)
Education		
Secondary, *n* (%)	1 (10)	2 (20)
Higher, *n* (%)	9 (90)	8 (80)
Employment status		
Unemployed, *n* (%)	1 (10)	0
Employed, *n* (%)	4 (40)	8 (80)
Retiree, *n* (%)	4 (40)	2 (20)
Student, *n* (%)	1 (10)	0
Smoker, *n* (%)	6 (60)	8 (80)
Comorbidities		
Respiratory disease, *n* (%)	3 (30)	2 (20)
Hypertension, *n* (%)	2 (20)	2 (20)
Cardiovascular disease, *n* (%)	2 (20)	0
Thyroid disease, *n* (%)	4 (40)	1 (10)
Severity of COVID-19 illness		
Mild/Moderate, *n* (%)	7 (70)	5 (50)
Severe, *n* (%)	1 (10)	4 (40)
Critical, *n* (%)	2 (20)	1 (10)
Hospitalization in clinic, *n* (%)	2 (20)	3 (30)
Hospitalization in clinic, days M (SD)	11.75 (6.23)	11.50 (6.19)
Hospitalization in ICU, *n* (%)	2 (20)	1 (10)
Hospitalization in ICU, days M (SD)	50.50 (10.60)	20.00 (0.00)
COVID-19 Symptoms		
Headache, *n* (%)	5 (50)	4 (40)
Muscle weakness, *n* (%)	10 (100)	10 (100)
Dyspnea, *n* (%)	6 (60)	7 (70)
Fatigue, *n* (%)	10 (100)	10 (100)
Chest pain, *n* (%)	5 (50)	7 (70)
Cough, *n* (%)	7 (70)	3 (30)
Long COVID-19 Syndrome symptoms		
Headache, *n* (%)	1 (10)	3 (30)
Muscle weakness, *n* (%)	7 (70)	7 (70)
Dyspnea, *n* (%)	7 (70)	8 (80)
Fatigue, *n* (%)	6 (60)	8 (80)
Memory/Concentration problems, *n* (%)	6 (60)	4 (40)
Pulmonary symptoms, *n* (%)	3 (30)	1 (10)
Cardiac symptoms, *n* (%)	5 (50)	6 (60)
Gastrointestinal symptoms, *n* (%)	2 (20)	4 (40)

**Table 2:** Differences between the pre-post measurements on muscular strength rise during 60 s sit-to-stand test and on dyspnea in the exercise group.

Variable	Exercise Group *n* = 10 M (SD)		
	Baseline	8 Weeks	*p*-Value
Muscle strength of right quadricep, kg	28.06 (5.91)	33.92 (6.49)	^a^ 0.03 *
Muscle strength of left quadricep, kg	26.06 (6.79)	34.32 (7.58)	^a^ 0.01 *
Rises at 60 s	24.10 (7.18)	27.80 (4.13)	^a^ 0.01 *
Dyspnea at the end of 60 s STS	2.50 (1.65)	1.55 (0.89)	^b^ 0.02 *

Note: Data are Mean Difference and Standard Deviation, MD (SD). Abbreviations: 60 s STS: 60 s Sit-To-Stand test. ^a^ Significant group effect at post-test (Paired *t*-test *p*  <  0.05). ^b^ Significant group effect at post-test (Wilcoxon Signed Ranks test) (* *p*  <  0.05).

**Table 3:** Differences between the pre-post measurements on muscular strength rise during 60 s sit-to-stand test and on dyspnea in the control group.

Variable	Control Group *n* = 10 M (SD)		
	Baseline	8 Weeks	*p*-Value
Muscle strength of right quadricep, kg	28.38 (6.08)	32.10 (11.21)	0.15
Muscle strength of left quadricep, kg	28.41 (9.75)	30.45 (9.58)	0.25
Rises at 60 s	22.40 (3.37)	26.10 (5.95)	^a^ 0.01 *
Dyspnea at the end of 60 s STS	2.25 (1.58)	1.80 (1.22)	0.16

Note: Data are Mean Difference and Standard Deviation, MD (SD). Abbreviations: 60 s STS: 60 s Sit-To-Stand test. ^a^ Significant group effect at post-test (Paired *t*-Test) (* *p*  <  0.05).

## Data Availability

Data available on request from the corresponding author.
